# Comparative genomics of vertebrate Fox cluster loci

**DOI:** 10.1186/1471-2164-7-271

**Published:** 2006-10-24

**Authors:** Karl R Wotton, Sebastian M Shimeld

**Affiliations:** 1 Department of Zoology, University of Oxford, The Tinbergen Building, South Parks Road, Oxford, OX1 3PS, UK

## Abstract

**Background:**

Vertebrate genomes contain numerous duplicate genes, many of which are organised into paralagous regions indicating duplication of linked groups of genes. Comparison of genomic organisation in different lineages can often allow the evolutionary history of such regions to be traced. A classic example of this is the Hox genes, where the presence of a single continuous Hox cluster in amphioxus and four vertebrate clusters has allowed the genomic evolution of this region to be established. Fox transcription factors of the C, F, L1 and Q1 classes are also organised in clusters in both amphioxus and humans. However in contrast to the Hox genes, only two clusters of paralogous Fox genes have so far been identified in the Human genome and the organisation in other vertebrates is unknown.

**Results:**

To uncover the evolutionary history of the Fox clusters, we report on the comparative genomics of these loci. We demonstrate two further paralogous regions in the Human genome, and identify orthologous regions in mammalian, chicken, frog and teleost genomes, timing the duplications to before the separation of the actinopterygian and sarcopterygian lineages. An additional Fox class, FoxS, was also found to reside in this duplicated genomic region.

**Conclusion:**

Comparison of loci identifies the pattern of gene duplication, loss and cluster break up through multiple lineages, and suggests *FoxS1 *is a likely remnant of Fox cluster duplication.

## Background

The Fox genes are members of the forkhead/winged helix family of transcription factors, characterised by a 110 amino acid DNA binding domain [1]. Fox genes have been identified in the genomes of animals and fungi, but not plants. Animals appear to have more Fox genes than fungi, with four genes identified in *Saccharomyces *and *Schizosaccharomyces*, at least 15 in the cnidarian sea anemone *Nematostella vectensis*, 17 in *Drosophila melanogaster*, 29 in *Ciona intestinalis *and over 40 in the human and other vertebrate genomes [1-4]. Phylogenetic analysis of the forkhead domains has lead to placement of most of these genes into 20 subclasses named *FoxA *to *FoxS*, with a small number of 'orphan' genes of unclear relationships defying classification [5,6]. These studies show vertebrate genomes contain more Fox genes than the genomes of other animals, suggesting an expansion that mirrors the increase in gene numbers seen for other gene families [7]. Two competing theories for how this increase in gene number took place are that two whole genome duplications (WGD; referred to as the 2R hypothesis) occurred early in the vertebrate lineage [8,9], or that continuous gene duplications occurred throughout vertebrate evolution [10-14]. The 2R hypothesis predicts that a 1:4 ratio of genes should have been present in an ancestral vertebrate when compared to an invertebrate, and that in molecular phylogenetic trees these paralogues would adopt a specific topology reflecting the history of duplication [11]. While the identification of blocks of paralogous genes has been interpreted as evidence in favour of WGD [15-24], paralagous gene sets often adopt different topologies [11], and multiple independent duplications could also explain the observed data.

Previously it has been reported that the human representatives of four Fox subclasses, *FoxC, FoxF, FoxL1 *and *FoxQ1*, are localised to two small regions of the human genome; specifically, the genes *FOXC2, FOXF1 *and *FOXQ1 *are found on chromosome 6 within 70 kb, while the genes *FOXL1, FOXC1, FOXF2 *are found on chromosome 16 within 300 kb [1,25,26]. Recently, we have shown that the amphioxus orthologues of these genes are found clustered in one region of the amphioxus genome, suggesting block duplication of this region in the vertebrates underlies the evolution of the chromosome 6 and 16 loci [25]. Under the 2R hypothesis, however, four such loci would be predicted. This discrepancy could be explained by gene loss.

To investigate this, we carried out a detailed comparative genomic survey of the human chromosome 6 and 16 Fox cluster loci. Genes around each locus were identified, and their evolutionary history established by database searches and molecular phylogenetics. This revealed two additional human loci containing genes paralagous to those found adjacent to the clustered Fox genes. All four loci were then compared to the genomes of other vertebrates, including other mammals and *Xenopus tropicalis, Gallus gallus, Fugu rubripes, Danio rerio *and *Tetraodon nigroviridis*. This allowed us to identify orthologous regions in all these animal genomes, and to show teleost genomes have undergone further duplications. By comparing these loci, we are now able to establish the pattern of clustered Fox gene duplication and loss through multiple vertebrate lineages. Furthermore, a previously orphan gene, *FoxS1*, was identified as a likely remnant of cluster duplication.

## Results

### Identification of human Fox cluster paralogons

**Figure 1:**
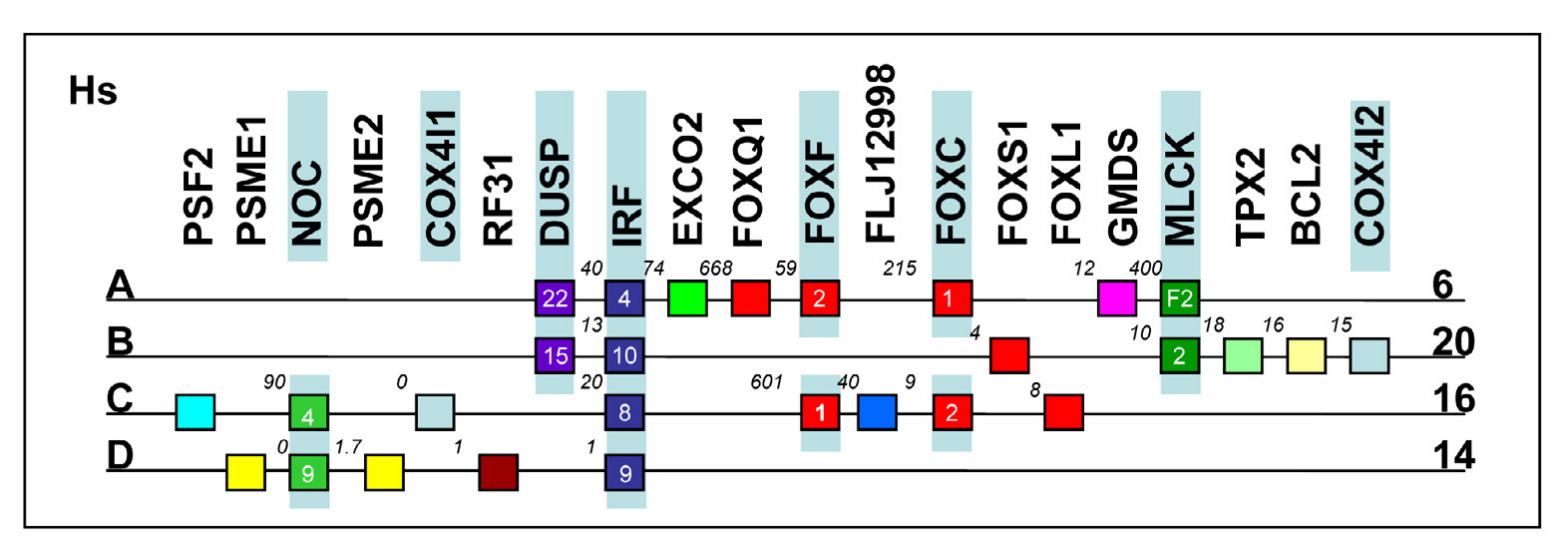
**Human paralogons of the FOX cluster loci**. A schematic representation of paralogy deduced from analysis of the human genome using NCBI map viewer Hs Build 36.1 (Nov 01, 2005) [36]. Gene names are shown above each gene, while numbers in the boxes indicate subfamily. Letters on the far left indicate paralogon designation, while numbers on the far right indicate which chromosome the cluster is found on. Numbers between genes are approximate intergenic distances in Kb. Background shading indicates paralogy.

Gene maps pertaining to this section are summarised in Figure 1. Our searches identified 5 gene families that showed evidence of genomic co-localisation with the Fox clusters; the Neighbour of COX (NOC) genes, the Cytochrome C Oxidase Subunit 4 (COX4) genes, the Dual Specificity Phosphatase (DUSP) genes, the Interferon Regulatory Factor (IRF) genes and the Myosin Light Chain Kinase (MLCK) genes. Phylogenetic analyses of these families are shown in Additional Files 1 and 2. To summarise, the NOC genes are found on the chromosome 16 and 14 (*NOC4 *and *NOC9*) and the COX4 genes are found on chromosome 16 and 20 (*COX4I1 *and *COX4I2 *respectively) though their exact positions relative to the other genes differ. DUSP genes are found next to the chromosome 6 and 20 clusters (*DUSP22 *and *DUSP15*). Each locus on chromosome 6, 20, 16 and 14 contains an IRF gene (*IRF4, IRF10, IRF8 *and *IRF9 *respectively). The *MLCK *genes are found by the chromosome 6 (*MLCKF2*) and 20 (*MLCK2*) clusters while a third gene, *MLCKF1 *is found on chromosome 16, though this last gene is not tightly linked to the rest of the cluster (and hence is not shown on Figure 1). Furthermore, another Fox gene, *FOXS1*, which has been previously regarded as an orphan [5,6], was identified adjacent to *IRF10 *on chromosome 20. We therefore conclude regions of chromosomes 14 and 20 shown in Figure 1 are the likely remaining paralogous genomic regions (paralogons) of the chromosome 6 and 16 Fox cluster regions.

### Orthologous paralogons in mammals, chicken, *Xenopus *and teleosts

**Figure 2:**
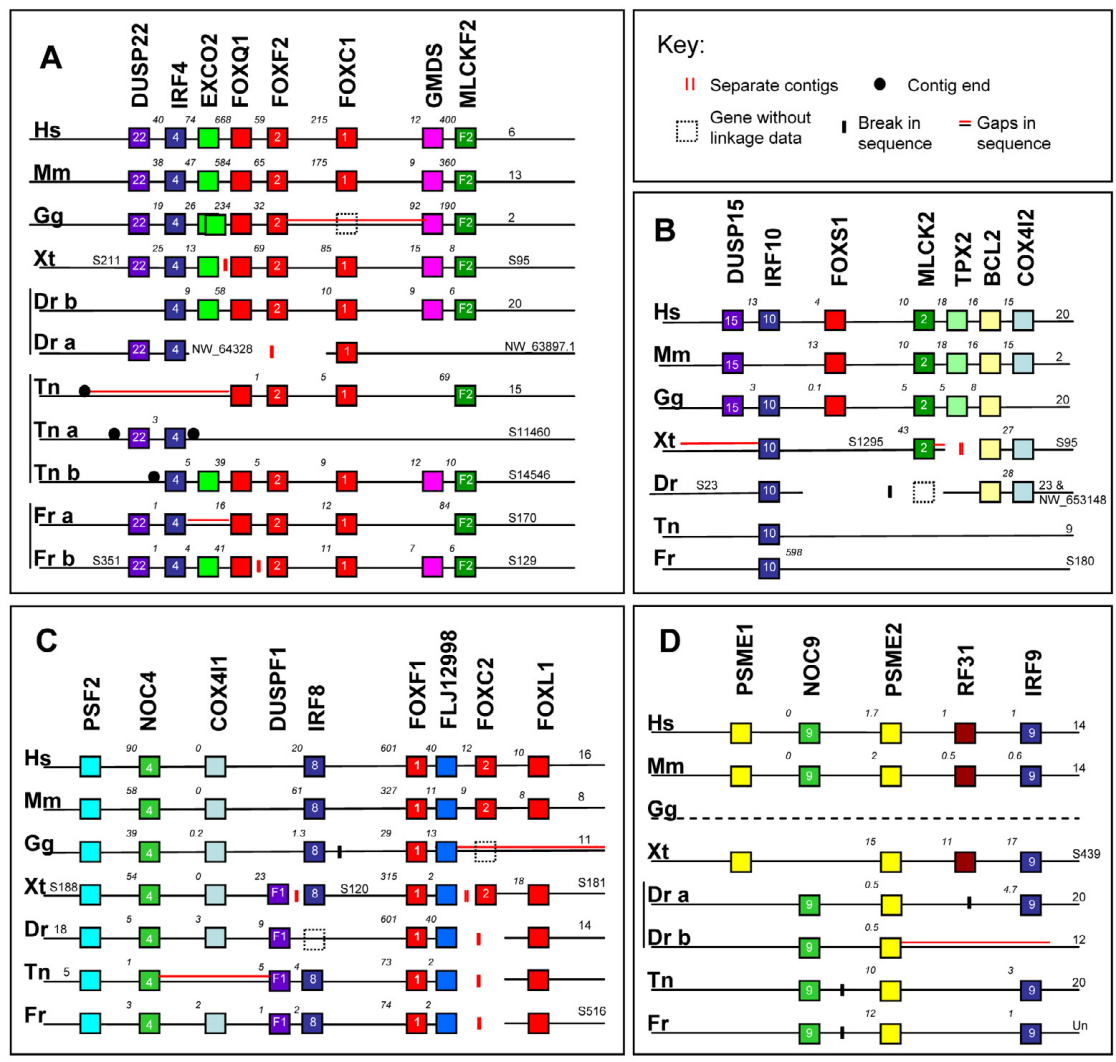
**Genomic organisation of human Fox cluster paralogons and putative orthologous counterparts**. Genomic organisation of human (Hs) Fox cluster paralogons and the putative orthologous counterparts we have identified in *Mus musculus *(Mm), *Xenopus tropicalis *(Xt), *Gallus gallus *(Gg), *Danio rerio *(Dr), *Tetraodon nigroviridis *(Tn) and *Fugu rubripes *(Fr). Panels A, B, C and D depict orthologous genomic regions to the human regions on chromosomes 6, 20, 16 and 14 respectively, as shown in Figure 1. This is a schematic diagram and not to scale. Colour coding indicates orthologous genes inferred by molecular phylogenetics (see additional files). Numbers at the ends of each line indicate chromosome number, or, where this is not available, scaffold number, with the latter indicated by S or NW. Numbers above lines indicate approximate distance in Kb between genes. A parallel red line indicates gaps in the sequence, while breaks in the sequence are indicated by double vertical black lines at the site of inversions. Double vertical red lines indicate separate contigs that we placed on a line due to their gene content; this does not imply they have been shown to be physically linked. Dashed boxes represent presence of a gene but no linkage information. Black circles indicate scaffold ends.

Conserved genomic regions to the four human loci described above were identified in the *M. musculus, X. tropicalis, G. gallus, F. rubripes, D. rerio *and *T. nigroviridis *genomes. These are summarised in Figure 2. We also examined chimp, dog and cow genomes. Gene organisation appeared similar to that in mouse and human (within the limits of the quality of genome assembly for these species; data not shown), and hence we have omitted them from the following sections. Further details of the phylogenetic analyses that confirm orthology and paralogy relationships are shown in the additional files that accompany this report. Below, we summarise the salient features of each orthologous set. In addition to the paralagous genes described in humans above, we also utilised other genes present in these genomic regions to re-enforce evidence of orthology.

### Genomic regions orthologous to human chromosome 6

This region consists of *DUSP22, IRF4, EXCO2, FOXQ1, FOXF2, FOXC1, GMDS *and *MLCKF2*. Genomic regions orthologous to human chromosome 6 are summarised in Figure 2A. A region of mouse chromosome 13 (and of chimp chromosome 6 and dog chromosome 35, data not shown) showed the order and intergenic distance of these genes to be comparable in these mammals. In the chicken, a region of chromosome 2 is orthologous to the same region, and includes the linked *FOXF2 *and *FOXQ1 *genes. A chicken *FOXC1 *gene was identified in a previous study [27], however its genomic position is currently unknown. We also note a large gap in the sequence between chicken *FOXF2 *and *GMDS*, and suggest chicken *FOXC1 *may reside here. In *X. tropicalis *this region is contained on two scaffolds; *dusp22, irf4 *and *exco5 *are on scaffold 211, and *foxq1, foxf2, foxc1, gmds *and *mlckf2 *on scaffold 95. High quality sequence is present for 150 kb and 230 kb past the final genes on each respective scaffold. However, as the gap between *Exoc2 *and *FoxQ1 *can exceed 500 kb in mammals, this does not preclude the linkage of these regions in the *X. tropicalis *genome.

A region in the *D. rerio *genome was identified on chromosome 20, extending from *irf4 *to *mlckf2 *and included identical gene organisation but relatively small intergenic distances when compared to tetrapods. *foxq1, foxf2 *and *foxc1b *are present in the expected positions. An additional contig containing linked *irf4*, and *dusp22 *orthologs was also identified. In the *T. nigroviridis *genome, two Fox clusters orthologous to human chromosome 6 were identified, one on chromosome 15 (extending from *foxq1 *to *mlckf2*) and one on unassigned scaffold 14546. An additional unassigned scaffold contains *irf4a *and *dusp22*, and could also be part of the chromosome 15 region as sequence quality is low adjacent to *foxq1*. This is supported by the *F. rubripes *arrangement, in which the orthologous genes are found on one scaffold.

In summary, we found evidence for single genomic regions orthologous to the Fox cluster region of human chromosome 6 in tetrapods, while in teleost fish we found evidence for the presence of two such regions, in keeping with the additional genome duplication proposed to have occurred in this lineage [28-31].

### Genomic regions orthologous to human chromosome 20

This region consists of *DUSP15, IRF10, FOXS1, MLCK2, TPX2, BCL2 *and *COX4I2*. Genomic regions orthologous to human chromosome 20 are summarised in Figure 2B. A region of mouse chromosome 2 (and of chimp chromosome 20, cow chromosome 13 and dog chromosome 24, data not shown) showed the order and intergenic distance of these genes to be comparable in these mammals. An exception is that no *Irf10 *gene was identified in this region of the mouse genome. In the chicken a single orthologous region was identified on chromosome 20, and included a *FOXS1 *orthologue. However we were unable to identify a *COX4I2 *gene. In *X. tropicalis*, two scaffolds (1295 and 95) were identified as orthologous to this human region; these do not overlap in gene content, hence we infer they derive from one region of orthology. *foxs1 *was not found in the *X. tropicalis *genome.

Evidence for orthologous regions in teleost genomes is weaker. A tenuous orthology region may exist in the *D. rerio *genome, as *bcl2 *and *cox4 *genes exhibit tight linkage on chromosome 23. An *irf10 *orthologue is also on chromosome 23, but at 44.65 Mb from the other genes, this linkage is unlikely to be significant. In *T. nigroviridis *and *F. rubripes*, we were only able to identify *irf10 *orthologues.

### Genomic regions orthologous to human chromosome 16

This region contains *PSF2, NOC4, COX4I1, IRF8, FOXF1, FLJ12998, FOXC2 *and *FOXL1*. Genomic regions orthologous to human chromosome 16 are summarised in Figure 2C. Investigation of mammalian genomes showed regions of chromosome 8 of the mouse (and of chimp chromosome 16, cow chromosome 18 and dog chromosome 5, data not shown) to be comparable in gene order and intergenic distance to the genes on human chromosome 16. Chromosome 11 of the chicken genome has a region orthologous to human chromosome 16, comprising of *PSF2, NOC4, COX4I1, IRF8 *and *FOXF1*. However part of this region is inverted in comparison to mammalian genomes. Similarity past *FOXF1 *extends to a region containing *FLJ12998*, which is found between *FOXF1 *and *FOXC2 *in the human genome. A chicken *FOXL1 *is yet to be identified, however the sequence quality upstream of *FoxF1 *in the chicken genome is poor.

Orthologous regions in the *X. tropicalis *genome are recovered from three separate scaffolds: s188 containing *psf2, noc4, cox4I1 *and a *dusp *family member. s120 contains *irf8, foxf1 *and *flj12998 *and s181 contains the *foxc2 *and *foxl1 *genes. Truncation of scaffolds 188 and 120 at the *irf8 *end do not preclude close linkage, however good sequence quality past *flj12998 *and before *foxc2 *suggest these regions, if linked, are minimally 3.5 Mb apart.

In *D. rerio*, an orthologous region on chromosome 18 matches the human arrangement from *psf2 *to a region with similarity to *flj12998 *with the exceptions that *D. rerio *(like *X. tropicalis *but unlike chicken and mammals) also has a *dusp *gene in this region, and that *irf8 *is absent and instead found on a contig yet to be assigned to a chromosome. Sequence quality past *foxf1 *appears to be high, however no *foxc2 *has been found in the genome of these teleosts. *foxc2 *is present in the basal Actinopterygian *A. calva *(see additional files), showing loss of *foxc2 *to be specific to teleosts. A *foxl1 *gene has been identified on chromosome 14. The arrangement in *T. nigroviridis *and *F. rubripes *is similar except *irf8 *is included in these clusters.

In summary, we find good evidence for orthologous regions to the human chromosome 16 Fox cluster region in the genomes of tetrapods and teleosts. However teleosts appear to have split this region between *flj12998 *and *foxl1*. An alternate explanation for this apparent split is they are remnants of the proposed teleost tetraplody, with reciprocal gene loss yielding non-overlapping gene sets. Higher quality genome assemblies will be needed to distinguish between these possibilities.

### Genomic regions orthologous to human chromosome 14

This region consists of *PSME1, NOC9, PSME2, RF31 *and *IRF9*. Genomic regions orthologous to human chromosome 14 are summarised in Figure 2D. Regions identified on mouse chromosome 14 (and on chimp chromosome 14, dog chromosome 8 and cow chromosome 10, data not shown), suggest preservation of this orthologous region in mammals. We were unable to identify a similar orthologous region (or any of the genes it contains) in the chicken genome, however a similar region was identified in the genome of *X. tropicalis*, suggesting the chicken has either lost these genes, or they are not included in the current assembly and associated sequence data. The *D. rerio *genome contains an *irf9, noc9, psme2 *orthology group on chromosome 20, with an inversion between *irf9 *and *psme2*. A second region on chromosome 12 includes a *noc9 *gene linked to a *psme2 *gene. The *T. nigroviridis *and *F. rubripes *genomes also contain a single region including *irf9, noc9 *and *psme2*, but with an inversion between *noc9 *and *psme2*.

## Discussion

### Human paralogons and orthologous regions in other vertebrates: gene duplication and gene loss

Here we report the results of a comparative analysis of vertebrate Fox cluster loci. First we identified four putative paralogons in the human genome; on chromosome 6 (with *DUSP22, IRF4, FOXQ1, FOXF2, FOXC1, MLCKF2 *linkage); on chromosome 20 (with *DUSP15, IRF10, FOXS1, MLCK2, COX4I2 *linkage); on chromosome 16 (with *NOC4, COX4I1, IRF8, FOXF1, FOXC2, FOXL1 *linkage); and on chromosome 14 (with *NOC9, IRF9 *linkage). Of these, that on chromosome 14 has the weakest supporting evidence, though IRF gene phylogenetics and the preservation of *NOC9 *and *IRF9 *linkage in other genomes supports its status as a paralagous region.

We also identified putative orthologous regions in the genomes of other vertebrates. Organisation in all mammals examined was very similar to that in human. In the tetrapods *G. gallus *and *X. tropicalis*, gene organisation was consistent with the ancestral tetrapod genome including a single copy of all four regions [32]. Some lineage specific changes are also inferred, and one paralogon is currently missing in *G. gallus*. The situation in the teleost genomes is more complex, with two putative orthologous regions identified for some of the loci. The teleost lineage is hypothesised to have undergone an additional genome duplication [28-31], and the pattern of orthologous regions we observed is consistent with this, though this would imply significant gene loss from several duplicated regions. Consistent with this we note that as high as 85% gene loss have been suggested for the teleosts following WGD [33].

### The evolutionary relationship of FoxS1

*FoxS1 *has previously been named in humans as *FKHL18 *[34] and in mouse as *Fkh3 *[35], however its phylogenetic relationship to Fox genes in more distant taxa has remained unresolved [5,6]. Our study extends the number of known *FoxS1 *genes to chimp, cow, dog and chicken orthologues, indicating the gene is at least as old as the mammal and bird lineage divergence. The consistent placement of *FoxS1 *in a genomic region paralagous to the clustered Fox genes suggests its evolutionary origin from the original Fox gene cluster. This is because the presence of orthologous regions in tetrapod and teleosts imply the origin of these paralogons, including the *FoxS1 *gene, via duplication prior to the separation of the actinopterygian and sarcopterygian lineages. Assuming this, it is possible the original pre-duplication Fox gene cluster included five genes (*FoxC, FoxL1, FoxF, FoxQ1 *and *FoxS1*), and that these have been fragmented by duplication and reciprocal gene loss. A literal interpretation of Fox gene phylogeny supports this view, as the *FoxS1 *genes group together, well separated from the other Fox subclasses. However, Fox gene phylogenies (including published accounts [5,6] and our study) are necessarily based on the relatively short sequence of the forkhead domain. Rapid divergence could lead to long branch attraction artefacts in such phylogenies, and hence we consider it possible that *FoxS1 *is paralagous to *FoxC, FoxL1, FoxF *or *FoxQ1*, and that this relationship has become obscured.

Furthermore, no *foxs1 *gene was recovered from the genomes of the teleosts or of *X. tropicalis*. We are currently unable to determine if this gene has been lost from these lineages, or is present but has not yet been sequenced. If the latter, the sequence quality in *X. tropicalis *and *T. nigroviridis *renders it unlikely it exists in the orthologous region of these genomes. This would raise a third possibility, that *FoxS1 *had independently translocated into this genomic region after the separation of the amphibian and amniote lineages. Sequencing of further vertebrate genomes may help resolve this issue.

### Fox cluster break-up in vertebrates

Retention of linkage between clustered *Fox *genes observed in humans, amphioxus and some insects has been hypothesised to have been constrained by co-ordinated regulation, analogous to that suggested for the homeobox genes [25]. Our results show the retention of Fox clusters in mammals. The chicken genome assembly is currently of insufficient quality to determine if the Fox clusters are intact, however in *X. tropicalis*, while *foxq1-foxf2-foxc1 *linkage is maintained, the *foxf1 *and *foxc2-foxl1 *genes are separated by at least 3.5 Mb, and could lie on different chromosomes.

In all three teleost genomes we found evidence for *foxq1-foxf2-foxc1 *linkage, however again the *foxf1-foxc2-foxl1 *cluster appears broken, and *foxc2 *appears to have been lost. In conclusion, our data do not disprove the possibility of constraint on the organisation of the *FoxQ1-FoxF2-FoxC1 *genes, but suggest such constraints, if they exist, are less likely on the organisation of the *FoxF1-FoxC2-FoxL1 *genes.

**Figure 3:**
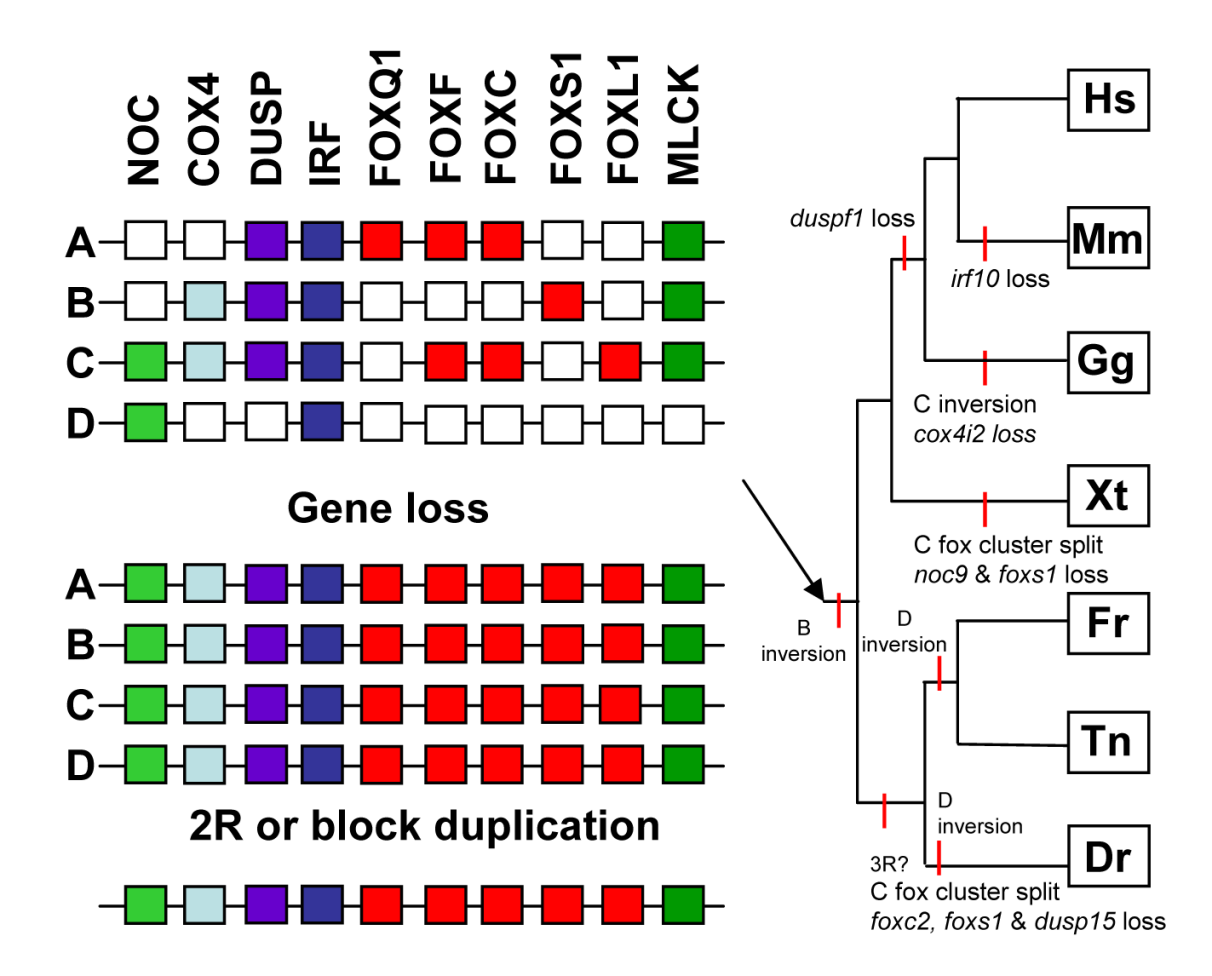
**A model for gene duplication and loss in the Fox cluster loci during vertebrate evolution**. Two initial rounds of duplication of the whole region are inferred to have occurred by the base of bony vertebrates, and this is consistent with both the 2R hypothesis and multiple block duplications. Considerable gene loss is then inferred before the radiation of bony vertebrates. Subsequent losses and inversions in different vertebrate lineages are indicated on the tree, with capital letters indicating the paralogon (nomenclature as shown in Figures 1 and 2) affected. A third round of genome duplication (3R) is indicated in the teleosts.

In summary, our data are consistent with the origin of paralagous regions via duplication of large regions of DNA. The timing of the duplications is consistent with that proposed for three of the genome duplications suggested to have occurred in vertebrate evolution, but also consistent with independent block duplication of this genomic region. Based on the pattern of duplication, we have constructed a model indicating how the Fox cluster locus evolved in vertebrates (Figure 3). Duplication of the whole region to yield four copies is inferred to have occurred by the base of bony vertebrates. Considerable gene loss is then inferred before the radiation of bony vertebrates. Subsequent losses and inversions in different vertebrate lineages are indicated in the figure.

## Conclusion

Comprehensive analysis of the human Fox cluster loci has identified 4 paralogons, 3 containing Fox genes and one Fox-less 'cryptic paralogon'. Extension of this comparison to other vertebrates has allowed a model for the evolution of this genomic region to be constructed, indicating patterns of gene duplication, loss, inversion and Fox cluster break up. To obtain a more complete picture of the evolution of the Fox clusters we note the importance of including basal actinopterygian, chondrichthian and agnathan data in future analyses to gain a greater understanding of the evolutionary history and timing of these events.

## Methods

Regions surrounding the human FOX cluster loci where searched using NCBI Map Viewer build 36.1 [36]. To identify further paralagous genes, BLAST searches [37] with putative protein sequences were conducted against the human genome, and putative positive targets further characterised by molecular phylogenetics to resolve orthologous and paralagous relationships. Each of the genes from the putative human paralogons was cross referenced by BLAST against the following NCBI genome assemblies; *Pan troglodytes *(build 1.1), *Bos taurus *(build 2.1), *Canis familiaris *(build 2.1), *Mus musculus *(build 3.5), *Gallus gallus *(build 1.1) and *Danio rerio *(Zv4) on NCBI map viewer. The *Xenopus tropicalis *and *Fugu rubripes *genomes were searched at the Joint Genome Institute genome portal [38], and the *Tetraodon nigroviridis *genome searched by BLAT via the Genoscope server [39]. Putative target sequences were further analysed by molecular phylogenetics, to establish orthologous and paralagous relationships and to reaffirm our original classification of human sequences. For molecular phylogenetic analyses, protein sequences were first aligned, (with invertebrate outgroup sequences included where possible) using ClustalX [40]. Phylogenetic analyses were carried out using Neighbor Joining and maximum likelihood implemented by ClustalX, and by PHYML [41,42].

## Authors' contributions

KRW conducted the database searches and phylogenetic analyses, and helped draft the manuscript. SMS helped draft the manuscript. Both authors read and approved the final manuscript.

## Supplementary Material

Additional File 1 **Gene family introduction and phylogeny interpretation**. Additional file 1 gives a brief introduction to each gene family, followed by a summary of the molecular phylogenetic analyses and their interpretation. This is followed by a table giving accession numbers or other identifiers for the sequences used in these analyses. A figure showing NCBI Map Viewer data of paralogous regions of the human genome is also included. Click here for file

Additional File 2 **Phylogenetic trees**. Phylogenetic trees of the Fox, MLCK, DUSP, COX, NOC and IRF families used in this analysis. Click here for file
