# Supplementary material for: Comparative genomics of vertebrate Fox cluster loci
Source: BMC Genomics. 2006 Oct 24;7:271. doi: 10.1186/1471-2164-7-271 (PMC1634998; doi:10.1186/1471-2164-7-271)
Supplement: Additional File 1 — Gene family introduction and phylogeny interpretation. Additional file 1 gives a brief introduction to each gene family, followed by a summary of the molecular phylogenetic analyses and their interpretation. This is followed by a table giving accession numbers or other identifiers for the sequences used in these analyses. A figure showing NCBI Map Viewer data of paralogous regions of the human genome is also included. [file 1471-2164-7-271-S1.pdf]

Additional file 1 – Gene family introduction and phylogeny interpretation

List of abbreviations

Ac	*Amia calva*
Am	*Apis mellifera*
Bf	*Branchiostoma floridae*
Bt	*Bos Taurus*
Ce	*Caenorhabditis elegans*
Ci	*Ciona intestinalis*
Cf	*Canis familiaris*
Dm	*Drosophila melanogaster*
Dp	*Drosophila pseudoobscura*
Dr	*Danio rerio*
Fr	*Fugu rubripes*
Gg	*Gallus gallus*
Hs	*Homo sapiens*
Mm	*Mus musculus*
Pt	*Pan troglodytes*
Rn	*Rattus norvegicus*
Sp	*Strongylocentrotus purpuratus*
Tn	*Tetraodon nigroviridis*
Xl	*Xenopus laevis*
Xt	*Xenopus tropicalis*

Below, we present a figure showing NCBI Map Viewer data of paralogous regions of the human genome. These data are summarised in Figure 1, and form the basis for its construction. We then give a brief introduction to each gene family discussed in the paper, followed by a summary of the molecular phylogenetic analysis and its interpretation. This is followed by a table giving accession numbers or other identifiers for the sequences used in these analyses. The trees resulting from the molecular phylogenetic analyses can be found in Additional File 2.

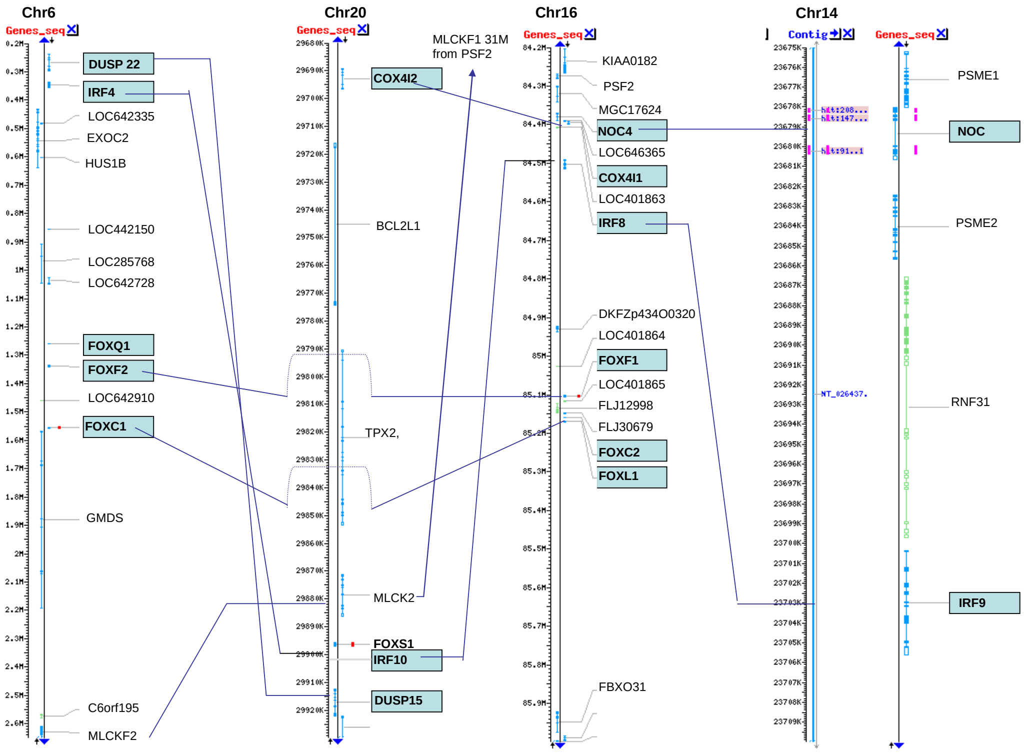

Figure 1 - Human paralogons of the FOX cluster loci. The views were generated by NCBI map viewer Hs Build 36.1 (Nov 01, 2005) by annotating genes onto genomic contigs. This includes known genes, and putative genes placed as a result of alignment of ESTs and mRNAs to the contigs. The view also includes BLAST hits found during this study. Regions of chromosomes (Chr) 6, 20, 16 and 14 are shown. The scale is in Mb for chromosomes 6 and 16, and in Kb for chromosomes 20 and 14. Lines connecting genes on different chromosomes indicate paralogy inferred by BLAST and subsequently confirmed by molecular phylogenetic analysis.

Gene Family introductions and analyses

The Fox gene family

FOXQ1 starts the Human 300kb chromosome 6 FOX cluster consisting of *FOXQ1*, *FOXF2* and *FOXC1* separated by 59kb and 215kb respectively (see Figure 1). Only one predicted gene appears in the cluster between *FOXF2* and *FOXC1*. e-PCR suggest LOC642910 be a pseudogene with similarity to *E74*-like factor 2 isoform 2. The Human chromosome 16 cluster spans 70kb with three genes found between *FOXF1* and *FOXC2*. The first, LOC401865 is a probable pseudogene with similarity to *60S ribosomal protein L7a*, the second, *FLJ12998*, is a novel gene with a conserved 5-formyltetrahydrofolate cyclo-ligase family domain. Homologous genes are also located next to *FoxF1* in mammals, *Gallus gallus*, *Xenopus tropicalis* and the teleost genomes. The third, *FLJ30679*, shows no homology to other genes. In this cluster *FOXC2* is 54kb from *FOXF1* and 10kb from *FOXL1*.

Mazet et al. (2003) resolved the phylogeny of the Fox gene family to show a close relationship between the *FoxL1* and *FoxC* subclasses and between the *FoxF* and *FoxQ1* subclasses [1]. The human gene *FREAC10* (now called *FOXS1*) resolved basally to the FoxC subclass in these analyses, but did not group with any other Fox genes.

Maximum likelihood analysis of the Fox genes *FoxF, FoxC, FoxL1, FoxQ1* and *FoxS1* was carried out using the forkhead domains of these genes. The tree was rooted using a human *FOXK1* sequence. All subfamilies include the amphioxus representative except for FoxS1 (where no gene has been identified).

The results essentially confirm previous studies [1] with the *FoxL1, FoxS1* and *FoxC* subfamilies most closely related to each other as are the *FoxQ1* and FoxF subfamilies. A previously *FoxC1* predicted chicken gene (automated computational analysis using GNOMON) branches as a *FoxS1* gene. Vertebrate *FoxF* and *FoxC* duplicates resolve within the FoxF and FoxC subfamilies as expected, however the high sequence identity between the duplicates in the forkhead domain reduces the resolution of these parts of the tree. To clarify this, separate trees were produced for each subset of genes using extended alignments. The Fox duplicates then resolved with high bootstrap values supporting the paralogy and synteny of the genomic regions identified in this study.

G. gallus FOXC1 was previously identified from a cDNA library [2] and is not represented in the FoxC tree due to doubtful 5' sequence. RACE used to extend the 5' end of the cDNA gave what is likely to be partial *FOXC2* sequence. Trees produced using more 3' regions of this gene confirm its placement as a *FOXC1***.** No *FoxC2* genes have been found in the genomes of teleost fish, however, in this study *foxc1* and *foxc2* genes have been cloned from *Amia calva,* a sister group to the teleosts [3] and are included in the *FoxC* tree. This finding suggests the *foxc2* loss to be teleost specific.

Primers used to clone *foxc1* and *foxc2* from *A. calva* genomic DNA.

**Forward**		M	Q	A	R	Y	S/P	V	
TetCfor	5'	ATG	CAG	GCN	MGN	TAY	HSN	GT	3'

**Reverse**		S	G	N	E/D	F	M	N	Y	
Lopfoxc2	5'	A	GCC	GTT	NTC	RAA	CAT	RTT	RTA	3'

Accession numbers for the *A. calva* sequences are AM402970 and AM402971

The IRF gene family

The IRF (interferon regulatory factor) family is a group of transcription factors that share homology in the DNA-binding domain [4, 5]. IRFs are mostly involved in the regulation of immune responses, in particular against viral infection. . It has been suggested the IRF genes arose early in vertebrate evolution from a prototypical protein with a *myb*-like DNA binding motif [6, 7]. Interestingly the *IRF* genes also encode a winged helix DNA binding domain with similarity to that encoded by the *Fox* genes, however the significance of this is unclear.

Maximum likelihood analysis of IRF sequences recovered previously described relationships among the IRF genes [6], and extended these due to expansion in the number and phylogenetic range of Irf sequences used. The tree is rooted with a *Strongylocentrotus purpuratus* (purple sea urchin) *Irf* sequence though it is unclear if this gene is an outgroup to all vertebrate IRF genes. Nehyba (2002) split the IRF

genes into four subfamilies, the IRF-1 subfamily contains the *Irf1* and *Irf2* genes, the IRF-5 subfamily contains the *Irf5* and *Irf6* genes, the IRF-3 subfamily contains the *Irf3* and *Irf7* genes and finally the IRF-4 subfamily contains the *Irf4*, *Irf*8, *Irf9* and *Irf10* genes as indicated on the tree.

Human *IRF10* has diverged to such an extent that it cannot produce a full length protein [6] and no mouse *Irf10* has been found (hence their omission from this analysis). However definitive *Irf10*-like sequences were recovered from the genomes of other mammals (not shown) and from other vertebrate genomes

Genes from the IRF-4 subfamily map to the genomic regions under consideration here, and in our analysis form a distinct group supported by a value of 85, indicating they are more closely related to each other than to the other IRF subfamilies. This supports our interpretation of *Irf4*, *Irf8*, *Irf9* and *Irf10* as paralagous genes derived from block duplications

The Dusp gene family

The Dusp (Dual specificity phosphatases) genes are a subclass of the protein tyrosine phosphatase (PTP) superfamily involved in the dephosphorylation of threonine and tyrosine residues in MAP kinase [8]. This is a large gene family, with over 20 representatives in the human genome. Maximum likelihood analysis of approximately 160 amino acids around the Dusp active site was used to produce the phylogenetic tree, which is rooted with a *C. elegans Dusp* sequence. Though some parts of the tree are supported by low bootstrap values, it does show the three genes under consideration here, *Dusp22*, *Dusp15* and *DuspF1,* to be closely related to each other, with a support value of 97. Sequences from *D. melanogaster and D. pseudoobscura* group basally to these genes suggesting them to be the orthologous to the vertebrate genes. A subfamily tree drawn using these genes resolves the relationship with high bootstrap values.

The COX4 gene family

COX4 (Cytochrome c oxidase subunit IV) forms part of the electron transport chain responsible for aerobic energy metabolism. Two types have been identified, named isoform 1 (I1) and isoform 2 (I2). In Humans *COX4I1* is located on chromosome 16 and shares a promoter with *NOC4* [9]; see below) while *COX4I2* is on chromosome 20. Expression analysis in rats has shown *Cox4I1* to be expressed ubiquitously, while *Cox4I2* shows high expression in adult lung with lower expression in all other tissue investigated [10]. No *Noc* related gene has been identified linked to *Cox4I2*. In the human genome a hypothetical protein (LOC646365) has been annotated overlapping with *COX4I1*, however this is likely a mis-annotation as some of the protein sequence encoded by this gene is that of the COX4I1 protein.

Cox4 orthologues were identified in *C. elegans, A. gambiae and S. purpuratus* and various vertebrates. Maximum likelihood analysis shows *Cox4I1* and *Cox4I2* to be paralogous genes with the duplication event producing them occurring somewhere between the sea urchin and teleost lineages. No *Cox4I2* was identified from the genomes of *G. gallus* or teleosts. The *cox4* gene found on chromosome 23 in the *D. rerio* genome falls outside both established vertebrate groups. As it is situated

44.65Mb from *irf10*, its presence of chromosome 23 cannot be considered informative.

The NOC4 gene family

NOC4 (Neighbour of COX4) is a novel gene of unknown function that shares its promoter with *COX4I1* on human chromosome 16 and whose expression appears to be ubiquitous [9]. Bachman et al (1999) found no significant homologies to known proteins, however, searches of the human genome in our study revealed a second *NOC* homologue 19kb from *IRF9* on chromosome 14 of the human genome, which we provisionally name here as *NOC9* signifying its linkage to *IRF9*.

NOC orthologues were identified in *C .elegans* (used to root the tree), *D. melanogaster, A. gambiae and S. purpuratus* and various vertebrates. Maximum likelihood analysis of these genes shows *Noc4* and *Noc9* to be paralogous genes with the duplication event producing them occurring somewhere between the sea urchin and teleost lineages. In *D. rerio* a third *noc* gene is found on chr12 and is not linked to an *irf9* gene. No *NOC9* or *IRF9* was found in the genome of the chicken.

The MLCK gene family

The MLCK (Myosin light chain kinase) gene family encodes enzymes involved in muscle contraction and myosin regulation [11-15].
Sequences were collected from *D. melanogaster* (known as *twichin*), *C. elegans* (known as *stretchin*) and various vertebrates. Maximum likelihood analysis of these sequences reveals three vertebrate gene groups. Mlck2 has been previously named, here we call the others *Mlckf1* and *Mlckf2* to signify their linkage to *FoxF1* and *FoxF2* respectively. However while *Mlckf1* is found linked to the *FoxF1-FoxC1-FoxL1* cluster in mammals and *G.gallus* it is several Mb distant. *Mlckf1* was not identified in the genomes of *X. tropicalis*, *F. rubripes* or *T. nigrovirdis*. The *D. rerio* gene has not been mapped . *Mlck2* is linked to Irf10 in amniotes but has not been mapped in *D.rerio* or identified in the other teleosts. The relationship of these vertebrate *Mlck* genes supports the paralogy and synteny of the genomic regions identified in this study.

Other genes

Several other genes for which molecular phylogenetics were not undertaken are shown in Figure 1. Molecular phylogenetics were not undertaken either because of insufficient sequence information, or more typically because there appears to be only one homolog associated with the paralagous gene families described above.

EXOC2. The exocyst complex component 2 protein is a component of the exocyst complex which targets exocytic vesicles to docking sites on the plasma membrane (Entrez gene). A blast search with overlapping LOC642335 protein hits EXOC2 in *P. troglodytes*.

HUS1B. HUS1 checkpoint homolog b (*S. pombe*). *HUS1B* is a paralog of human *HUS1* with suggested roles in regulating cell cycle checkpoints and genomic integrity [16].

GMDS. GDP-mannose 4,6-dehydratase is the first enzyme in the pathway converting GDP-mannose to GDP-fucose [17].

BCL. Members of the **BCL**-2 family act as anti or pro-apoptotic regulators and form hetro or homodimers involved in a wide variety of cellular activities (Entrez gene).

TPX2 is a microtubule-associated homologue with a role in spindle assembly [18].

PSF2. Partner of Sld five 2 is a component of the GINS multiprotein complex involved in the initiation of DNA replication [19-21]. A second PSF2-like gene is found on chromosome 20.

FBX031 is F-box protein 31 a ubiqitin ligase specificity factor [22].

PSME is proteasome activator subunit 1 and 2. These subunits are regulators of the immunoproteasome, an altered 26S proteasome that processes class I MHC peptides (Entrez gene).

RNF31 is ring finger protein 31. The gene contains a ring finger motif known to be involved in protein-protein and protein-DNA interactions (Entrez gene).

Novel genes

LOC442150, LOC285768, LOC642728, MGC17624, DKFZp43400320 are hypothetical genes without clear homologies to other genes.

Pseudogenes

LOC401863 is similar to ribosomal protein L10a, LOC401865 is similar to chloride intracellular channel 1. Pseudogenes are also found in each FOX cluster (detailed above).

References

1. Mazet F, Yu JK, Liberles DA, Holland LZ, Shimeld SM: **Phylogenetic relationships of the Fox (Forkhead) gene family in the Bilateria.** *Gene* 2003, **316:**79-89.
2. Buchberger A, Schwarzer M, Brand T, Pabst O, Seidl K, Arnold HH: **Chicken winged-helix transcription factor cFKH-1 prefigures axial and appendicular skeletal structures during chicken embryogenesis.** *Dev Dyn* 1998, **212:**94-101.
3. Kikugawa K, Katoh K, Kuraku S, Sakurai H, Ishida O, Iwabe N, Miyata T: **Basal jawed vertebrate phylogeny inferred from multiple nuclear DNA-coded genes.** *BMC Biol* 2004, **2:**3.

4. Harada H, Taniguchi T, Tanaka N: **The role of interferon regulatory factors in the interferon system and cell growth control.** *Biochimie* 1998, **80:**641-650.
5. Mamane Y, Heylbroeck C, Genin P, Algarte M, Servant MJ, LePage C, DeLuca C, Kwon H, Lin R, Hiscott J: **Interferon regulatory factors: the next generation.** *Gene* 1999, **237:**1-14.
6. Nehyba J, Hrdlickova R, Burnside J, Bose HR, Jr.: **A novel interferon regulatory factor (IRF), IRF-10, has a unique role in immune defense and is induced by the v-Rel oncoprotein.** *Mol Cell Biol* 2002, **22:**3942-3957.
7. Veals SA, Schindler C, Leonard D, Fu XY, Aebersold R, Darnell JE, Jr., Levy DE: **Subunit of an alpha-interferon-responsive transcription factor is related to interferon regulatory factor and Myb families of DNA-binding proteins.** *Mol Cell Biol* 1992, **12:**3315-3324.
8. Camps M, Nichols A, Arkinstall S: **Dual specificity phosphatases: a gene family for control of MAP kinase function.** *Faseb J* 2000, **14:**6-16.
9. Bachman NJ, Wu W, Schmidt TR, Grossman LI, Lomax MI: **The 5' region of the COX4 gene contains a novel overlapping gene, NOC4.** *Mamm Genome* 1999, **10:**506-512.
10. Huttemann M, Kadenbach B, Grossman LI: **Mammalian subunit IV isoforms of cytochrome c oxidase.** *Gene* 2001, **267:**111-123.
11. Teichmann SA, Chothia C: **Immunoglobulin superfamily proteins in Caenorhabditis elegans.** *J Mol Biol* 2000, **296:**1367-1383.
12. Champagne MB, Edwards KA, Erickson HP, Kiehart DP: **Drosophila stretchin-MLCK is a novel member of the Titin/Myosin light chain kinase family.** *J Mol Biol* 2000, **300:**759-777.
13. Potier MC, Chelot E, Pekarsky Y, Gardiner K, Rossier J, Turnell WG: **The human myosin light chain kinase (MLCK) from hippocampus: cloning, sequencing, expression, and localization to 3qcen-q21.** *Genomics* 1995, **29:**562-570.
14. Fong S, Hamill SJ, Proctor M, Freund SM, Benian GM, Chothia C, Bycroft M, Clarke J: **Structure and stability of an immunoglobulin superfamily domain from twitchin, a muscle protein of the nematode Caenorhabditis elegans.** *J Mol Biol* 1996, **264:**624-639.
15. Ayme-Southgate A, Vigoreaux J, Benian G, Pardue ML: **Drosophila has a twitchin/titin-related gene that appears to encode projectin.** *Proc Natl Acad Sci U S A* 1991, **88:**7973-7977.
16. Hang H, Zhang Y, Dunbrack RL, Jr., Wang C, Lieberman HB: **Identification and characterization of a paralog of human cell cycle checkpoint gene HUS1.** *Genomics* 2002, **79:**487-492.
17. Sullivan FX, Kumar R, Kriz R, Stahl M, Xu GY, Rouse J, Chang XJ, Boodhoo A, Potvin B, Cumming DA: **Molecular cloning of human GDP-mannose 4,6-dehydratase and reconstitution of GDP-fucose biosynthesis in vitro.** *J Biol Chem* 1998, **273:**8193-8202.
18. Garrett S, Auer K, Compton DA, Kapoor TM: **hTPX2 is required for normal spindle morphology and centrosome integrity during vertebrate cell division.** *Curr Biol* 2002, **12:**2055-2059.
19. Obama K, Ura K, Satoh S, Nakamura Y, Furukawa Y: **Up-regulation of PSF2, a member of the GINS multiprotein complex, in intrahepatic cholangiocarcinoma.** *Oncol Rep* 2005, **14:**701-706.

20. Takayama Y, Kamimura Y, Okawa M, Muramatsu S, Sugino A, Araki H: **GINS, a novel multiprotein complex required for chromosomal DNA replication in budding yeast.** *Genes Dev* 2003, **17:**1153-1165.
21. Kubota Y, Takase Y, Komori Y, Hashimoto Y, Arata T, Kamimura Y, Araki H, Takisawa H: **A novel ring-like complex of Xenopus proteins essential for the initiation of DNA replication.** *Genes Dev* 2003, **17:**1141-1152.
22. Jin J, Cardozo T, Lovering RC, Elledge SJ, Pagano M, Harper JW: **Systematic analysis and nomenclature of mammalian F-box proteins.** *Genes Dev* 2004, **18:**2573-2580.

Sequence accession and equivalent identification numbers

The tables below give accession numbers or equivalent for sequences used in molecular phylogenetic analyses. Tree name indicates the name used on the phylogenetic tree for the family. Location is the chromosome (Chr) or scaffold (S) number (if known). Databases are as follows
AN: Genbank: http://www.ncbi.nlm.nih.gov/
JGI: Joint Genome Institute: http://genome.jgi-psf.org/
E: Ensembl gene identifirl: http://www.ensembl.org/index.html
Hm: Gnomon model number on map viewer: http://www.ncbi.nlm.nih.gov/

MLCK sequences			
**Tree name**	**Location**	**ID Number**	**Database**
BtF1chr18	Chr18	XP_604749.2	AN
BtF2		XP_596207.2	AN
CeTwitchin		CAA33463.1	AN
DmStretchin		NP_523754.2	AN
Dr2		XP_687022.1	AN
DrF1		CAE49228.1	AN
DrF2aChr20	Chr20	XP_697572.1	AN
DrF2b		XP_695224.1	AN
FrF2as129	S129	604718	JGI
FrF2bs107	S107	580153	JGI
Gg2chr20	Chr20	NP_990723.1	AN
GgF1chr11	Chr11	XP_414113.1	AN
GgF2chr2	Chr2	XP_418978.1	AN
Hs2chr20	Chr20	NP_149109.1	AN
HsF1chr16	Chr16	AAI09097.1	AN
HsF2chr6	Chr16	CAI12220.1	AN
Mm2	Chr2	XP_130630.6	AN
MmF1chr8	Chr8	BAC35177.1	AN
MmF2chr13	Chr13	XP_915653.1	AN
Rr2		NP_476557.1	AN
TnF2bchr15	Chr15	GSTENP00016215001	E
XtF2s95	S95	329016	JGI

NOC sequences			
**Tree name**	**Location**	**ID Number**	**Database**
AgNOC		XP_554509.1	AN
BtNOCchr10	Chr10	XP_586913.2	AN
CeF25H2.4		NP_492760.1	AN
CfNOC4chr5	Chr5	XP_536760.2	AN
CfNOCchr8	Chr8	XP_537381.2	AN
DmNOC		NP_611731.1	AN
DrNOC4chr18	Chr18	NP_998535.1	AN
DrNOCchr12	Chr12	NP_956420.1	AN
DrNOCchr20	Chr20	XP_692545.1	AN
FrNOC4s96	S96	712068	JGI
FrNOCs14	S14	292321	JGI
GgNOC4chr11	Chr11	XP_414188.1	AN
HsNOC4chr16	Chr16	NP_006058.1	AN
HsNOCchr14	Chr14	NM_016049	AN
MmNOC4chr8	Chr8	NP_035056.1	AN
MmNOCchr14	Chr14	NP_149158.1	AN
SpNOC4		XP_787518.1	AN
TnNOCchr5	Chr5	GSTENP00017647001	E
TnNOCchrUn	ChrUn	GSTENT00016547001	E
XlCOX4		AAH63338.1	AN
XlNOC		AAI06463.1	AN
XtNOC4s188	S188	454231	JGI

COX4 sequences			
**Tree name**	**Location**	**ID Number**	**Database**
AgCox4		XP_557879.1	AN
BtI1chr18	Chr18	NP_001001439.1	AN
BtI2chr13	Chr13	XP_875763.1	AN
CeCOX4		NP_493394.1	AN
CfI1chr5	Chr5	XP_536759.1	AN
CfI2chr24	Chr24	XP_542949.2	AN
DrCOXchr23	Chr23	NP_957097.1	AN
DrI1chr18	Chr18	NP_999866.1	AN
FrI1S14	S14	749214	JGI
GgI1chr11	Chr11	NP_001025748.1	AN
HsI1chr16	Chr16	NP_001852.1	AN
HsI2chr20	Chr20	NP_115998.2	AN
MmI1chr8	Chr8	NP_034071.1	AN
Mmi2chr2	Chr2	NP_444321.1	AN
PtI1chr16	Chr16	XP_511235.1	AN
PtI2chr20	Chr20	XP_525298.1	AN
SpCOX4		XP_796538.1	AN
XtI1s188	S188	169573	JGI

COX4 sequences			
**Tree name**	**Location**	**ID Number**	**Database**
XtI2s95	S95	NP_001016945.1	AN

DUSP sequences			
**Tree name**	**Location**	**ID Number**	**Database**
AgDUSP		XP_320303.2	AN
CeDUSP		NP_501053.2	AN
Dm		NP_729909.1	AN
DmDUSP		NP_649087.1	AN
Dp		GA10063-PA	AN
Dr1		NP_998232.1	AN
Dr1522chr18	Chr18	NP_001002514.1	AN
Dr19		NP_998144.1	AN
Dr22chrUn	ChrUn	NW_643208.1	AN
Dr23		NP_001034709.1	AN
Dr4		NP_957465.1	AN
Dr5		NP_997730.1	AN
Dr7		AAH66600.1	AN
Fr1522s14	S14	749213	JGI
Fr22s107	S107	580074	JGI
Fr22s351	S351	576242	JGI
Frs180		605441	JGI
Gg10		NP_001026215.1	AN
Gg15chr20	Chr20	XP_417451.1	AN
Gg16		XP_428887.1	AN
Gg22chr2	Chr2	XP_418974.1	AN
Gg6		NP_989685.1	AN
Hs1		NP_004408.1	AN
Hs10		NP_009138.1	AN
Hs11		NP_003575.1	AN
Hs12		AAH06286.1	AN
Hs13		CAI40905.1	AN
Hs14		CAG46599.1	AN
Hs15chr20	Chr20	CAI12821.1	AN
Hs16		AAH42101.1	AN
Hs18		AAH30987.1|	AN
Hs19		AAH93958.1|	AN
Hs2		NP_004409.1	AN
Hs21		NP_071359.2	AN
Hs22Chr6	Chr6	NP_064570.1	AN
Hs23		NP_060293.2	AN
Hs24		Q9Y6J8	AN

DUSP sequences			
**Tree name**	**Location**	**ID Number**	**Database**
Hs26		AAH67804.1	AN
Hs27		XP_940528.1	AN
Hs3		AAH35701.1	AN
Hs4		NP_001385.1	AN
Hs5		NP_004410.3	AN
Hs6		NP_001937.2	AN
Hs7		NP_001938.1	AN
Hs8		AAH38231.1	AN
Hs9		NP_001386.1	AN
Mm1		NP_038670.1	AN
Mm10		AAH25066.1	AN
Mm11		AAH28640.1	AN
Mm12		AAH99453.1	AN
Mm13		AAI00422.1	AN
Mm14		NP_062793.2	AN
Mm15chr2	Chr2	NP_665687.1	AN
Mm16		AAH57321.1	AN
Mm18		AAH20036.1|	AN
Mm19		AAH21591.1|	AN
Mm2		AAH48696.1	AN
Mm21		NP_082844.1	AN
Mm22chr13	Chr13	NP_001033044.1	AN
Mm23		NP_081001.1	AN
Mm26		AAH18204.1	AN
Mm27		NP_00102851	AN
Mm3		Q9D7X3	AN
Mm4		NP_795907.1	AN
Mm6		NP_080544.1	AN
Mm7		NP_703189.1	AN
Mm8		AAH52705.1	AN
Mm9		NP_083628.3	AN
Rn24		AAH98678.1	AN
Rn5		NP_598262.1	AN
Tn1522chr5	Chr5	GSTENT00017648001	E
Tn22chrUn	ChrUn	GSTENT00009638001	E
Xt1522s188	S188	201026	JGI
Xt22s211	S211	170578	JGI

IRF sequences			
**Tree name**	**Location**	**ID Number**	**Database**
Bt10chr13	Chr13	hmm248044	HM
Bt4chrUn	ChrUn	XP_870405.1	AN
Bt8Chr18	Chr18	hmm42785	HM
Bt9Chr10	Chr10	AAX46743.1	AN
Cf10chr24	Chr24	hmm45655	HM
Cf4chr35	Chr35	hmm314	HM
Cf8chr5	Chr5	hmm37927	AN
Dr1?		AAQ97797.1	AN
Dr10chr23	Chr23	NM_212879	AN
Dr3chr12	Chr12	XP_693269.1	AN
Dr3chr15	Chr15	XP_694284.1	AN
Dr4achrUn	ChrUn	AF320331_1	AN
Dr4bChr20	Chr20	hmm79540	HM
Dr8chrUn	ChrUn	NM_001002622	AN
Dr9chr20	Chr20	NM_205710	AN
DrIRF6		AAH56772.1	AN
DrIRF7		AAH65902.1	AN
Fr10s180	S180	605425	JGI
Fr4aS107	S107	580063	JGI
Fr4s351	S351	576245	JGI
Fr8s14	S14	749212	JGI
Fr9s96	S96	613411	JGI
Gg1		NP_990746	AN
Gg10chr20	Chr20	NP_989889.1	AN
Gg2		CAA64748.1	AN
Gg3?		Q90643	AN
Gg4chr2	Chr2	NP_989630.1	AN
Gg5		CAG32068.1	AN
Gg8chr11	Chr11	Q90871	AN
Hs1		NP_002189.1	AN
Hs2		NP_002190.1	AN
Hs3		Q14653	AN
Hs4chr6	Chr6	AAH15752.1	AN
Hs5		Q13568	AN
Hs7		NP_001563.2	AN
Hs8chr16	Chr16	Q02556	AN
Hs9Chr14	Chr14	Q00978	AN
Mm1		NP_032416.1	AN
Mm2		NP_032417.2	AN
Mm3		P70671	AN

IRF sequences			
**Tree name**	**Location**	**ID Number**	**Database**
Mm4chr13	Chr13	CAI24563.1	AN
Mm5		P56477	AN
Mm7		NP_058546.1	AN
Mm8chr8	Chr8	NP_03234	AN
Mm9chr14	Chr14	AAC52494.1	AN
Om?		AF332147_1	AN
SpIRF		hmm23186	HM
SsIRF6		NP_999443.1	AN
Tn10	Chr9	GSTENT00023713001	E
Tn4a	S11460	GSTENT00009637001	E
Tn4b	S14546	GSTENP00015662001	E
Tn8chr5	Chr5	GSTENT00017649001	E
Tn9		GSTENT00016546001	E
Tr1?		AF242447_1	AN
Xl1		NP_001006695.1	AN
Xt10s1295	S1295	ENSXETP00000005434	E
Xt2		NP_001008014.1	AN
Xt4s211	S211	349993	JGI
Xt8s120	S120	334257	JGI
Xt9s439	S439	375197	JGI
XtIRF6		NP_001025493.1	AN

Fox sequences			
**Tree name**	**Location**	**ID Number**	**Database**
AcC1			
AcC2			
Bf		AY9236	AN
BfC		CAH69694	AN
BfF		CAH69695	AN
BfL1			
BfQ1		CAH55831	AN
BtS1	Chr13	hmm247650	HM
CfS1	Chr24	hmm45519	HM
CiF		BAE06437.1	AN
DrC1a	ChrUn	NM_131728	AN
DrC1b	Chr20	NM_131729	AN
DrF1	Chr18	XP_694768.1	AN
DrF2Chr20		hmm35595	HM
DrL1	Chr14	NM_200984	AN
DrQ1	Chr20	AAH67659	AN
FrC1s107	s107	580056	JGI

Fox sequences			
**Tree name**	**Location**	**ID Number**	**Database**
FrC1s129	S129	604756	JGI
FrF1s14	S14	611269	JGI
FrF2s107	S107	580109	JGI
FrF2s129	S129	604700	JGI
FrL1	s516	165281	JGI
FrQ1s107	S107	580109	JGI
FrQ1s351	S351	576230	JGI
GgC1 (cfkh-1)		NP_990337	AN
FOXS1 (previously GgC1)	Chr2	XP_427911.1	AN
GgC2chrUn	ChrUn	NP_990469.1	AN
GgF1	Chr11	XP_414186.1	AN
HsC1	Chr6	NM_001453	AN
HsC2	Chr16	NM_005251	AN
HsF1	Chr16	NM_001451	AN
HsF2		NM_001452	AN
HsK1		NP_00103224	AN
HsL1	Chr16	NM_005250	AN
HsQ1	Chr6	NP_150285.2	AN
HsS1	Chr20	CAI12817.1	AN
MmC1	Chr13	CAI25767	AN
MmC2	Chr8	NP_038547	AN
MmF2	Chr13	CAI25803	AN
MmL1		XP_921996.1	AN
MmQ1	Chr13	NM_008239	AN
MmS1	Chr2	NP_034356	AN
PtS1	Chr20	hmm7964	HM
SpC		ABB89478.1	AN
SpL1		XP_784909.1	AN
SpQ1		ABB89489.1	AN
TnC1chr15	Chr15	GSTENP00016221001	E
TnC1s14546	s14546	GSTENT00015666001	E
TnF1	Chr5	GSTENP00017651001	E
TnF2chr15	Chr15	GSTENT00016222001	E
TnF2s14546	ChrUn	GSTENT00015665001	E
TnQ1chr15	Chr15	GSTENP00016223001	E
TnQ1s14546		GSTENP00015665001	E
XtC1	s95	NM_001007863	AN
XtC2	s181	NM_213692	AN
XtF1		476259	JGI
XtF2s95	S95	91457	JGI
XtL1		345221	JGI
XtQ1		ABA39837	AN
