# Supplementary material for: Comparative genomics of vertebrate Fox cluster loci
Source: BMC Genomics. 2006 Oct 24;7:271. doi: 10.1186/1471-2164-7-271 (PMC1634998; doi:10.1186/1471-2164-7-271)

Cox4 maximum likelihood tree

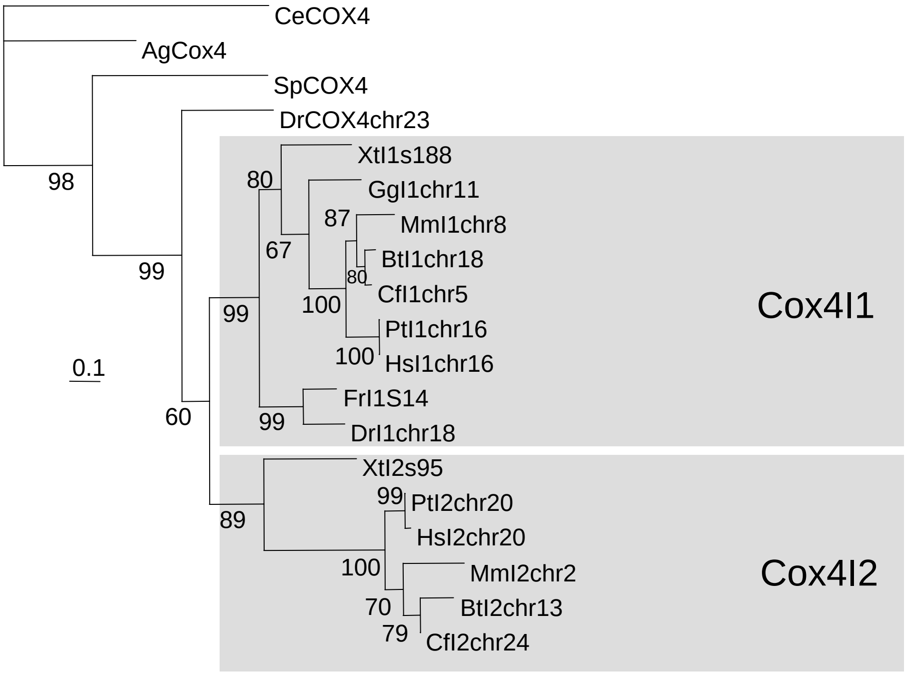

Noc maximum likelihood tree

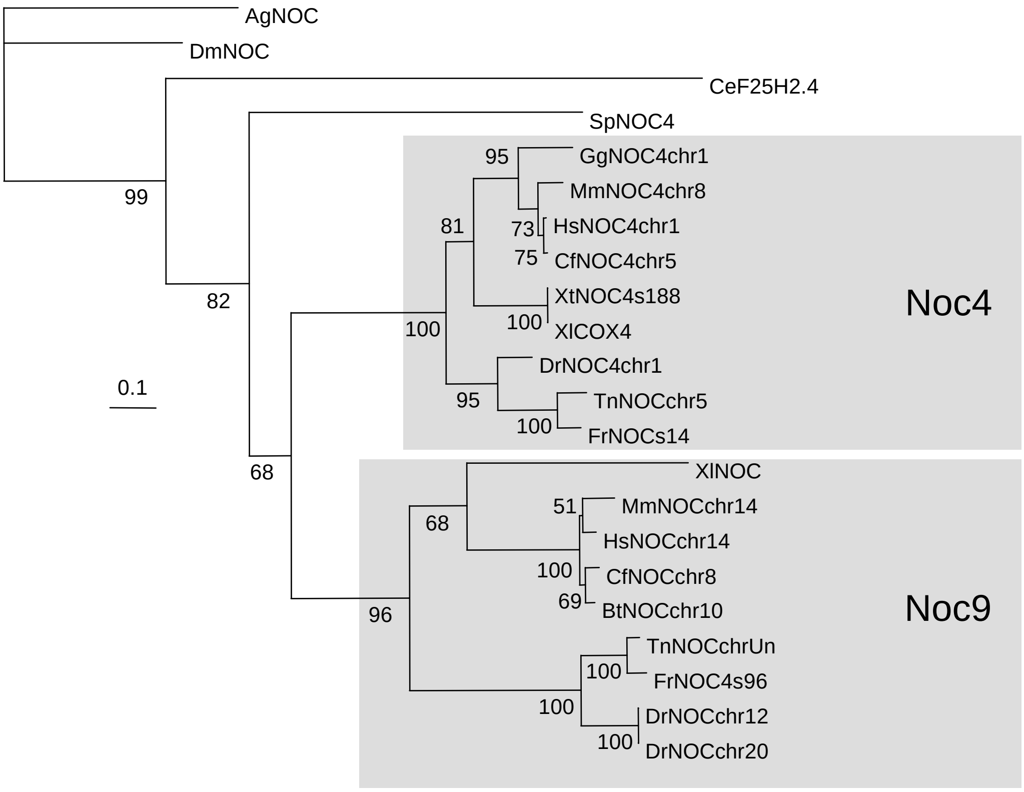

Mlck maximum likelihood tree

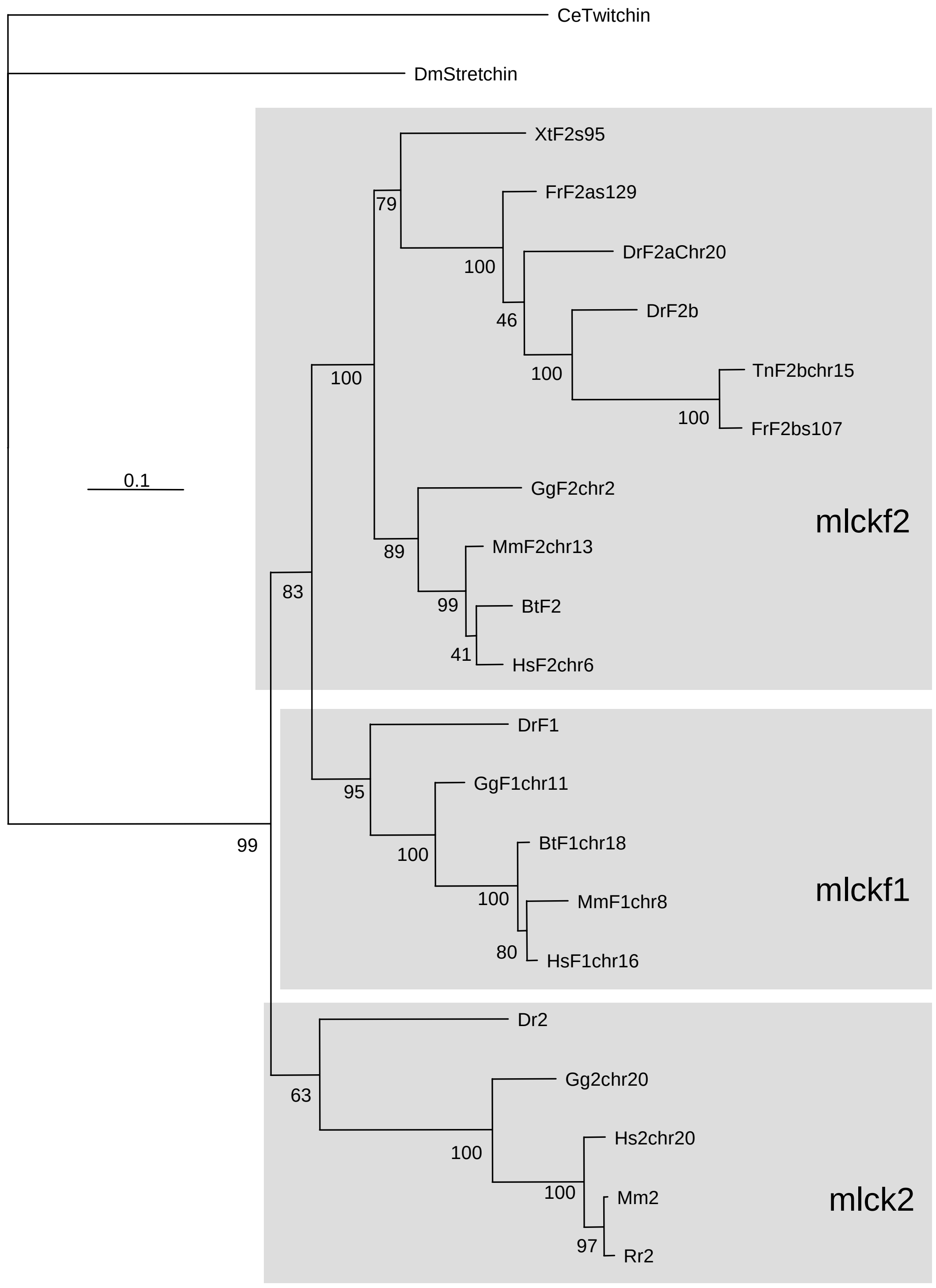

Irf family maximum likelihood tree

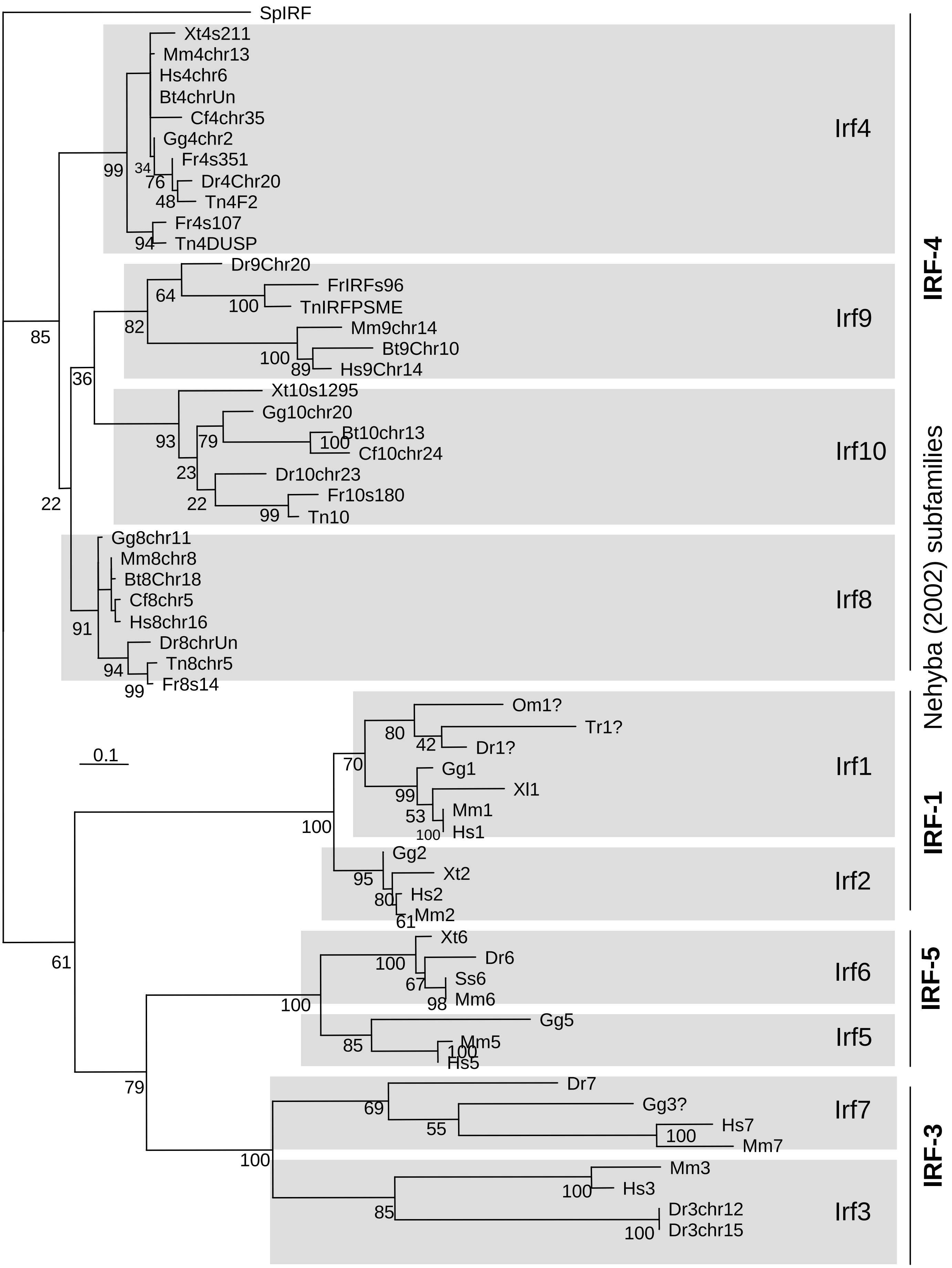

FoxF, FoxQ1, FoxL1, FoxC and FoxS1 subfamily neighbour joining tree

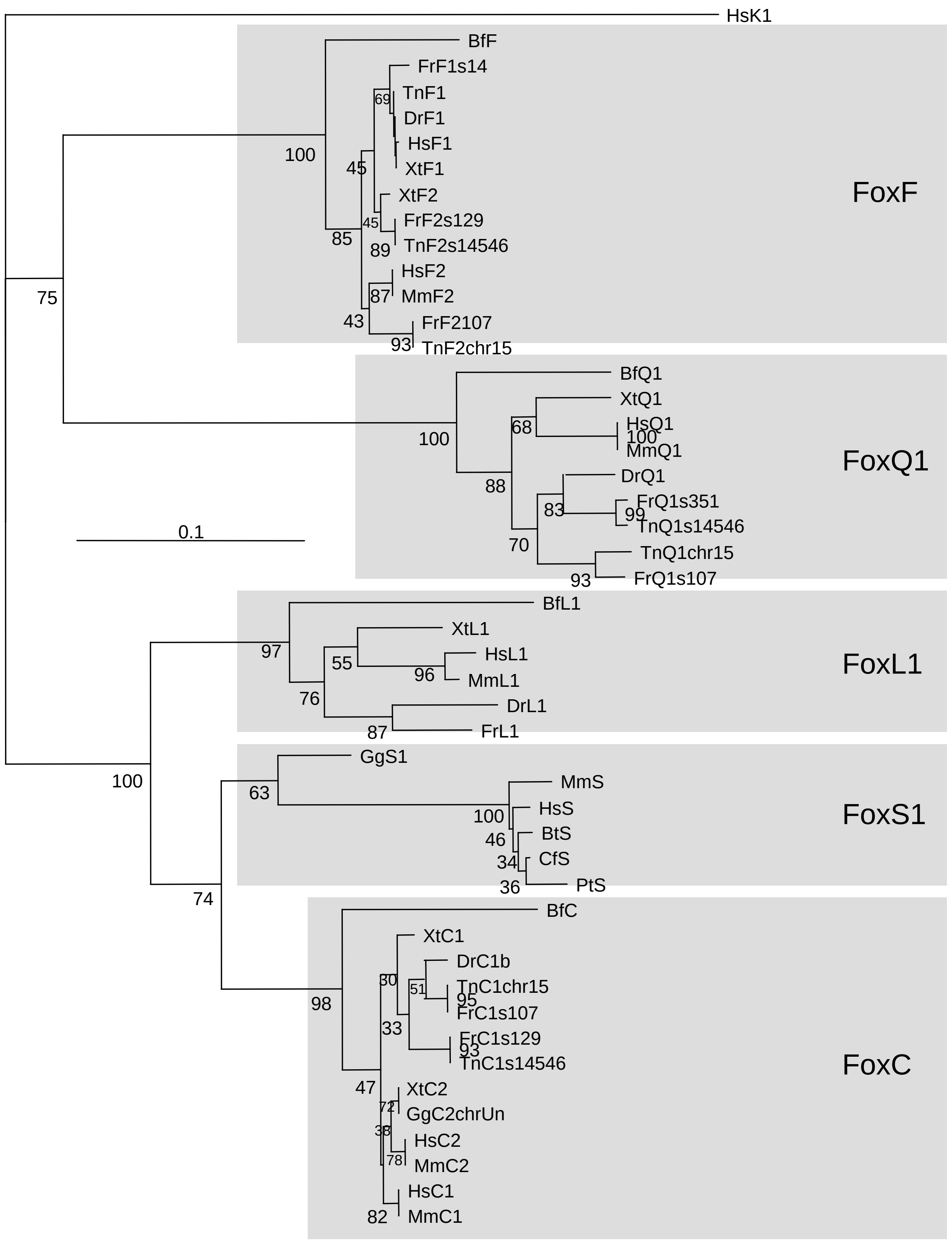

FoxF, FoxC, FoxQ1, FoxL1 subfamily extended alignment maximum likelihood trees

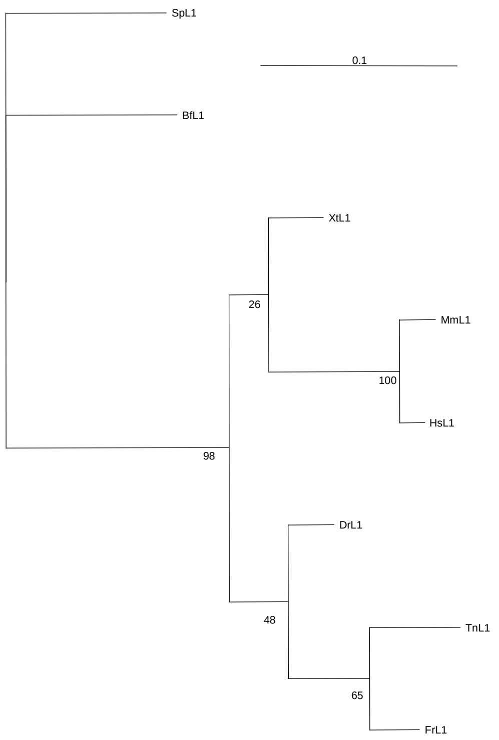

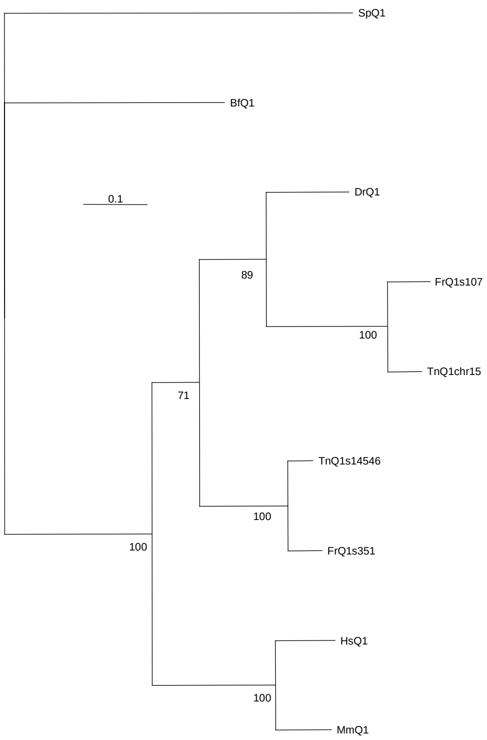

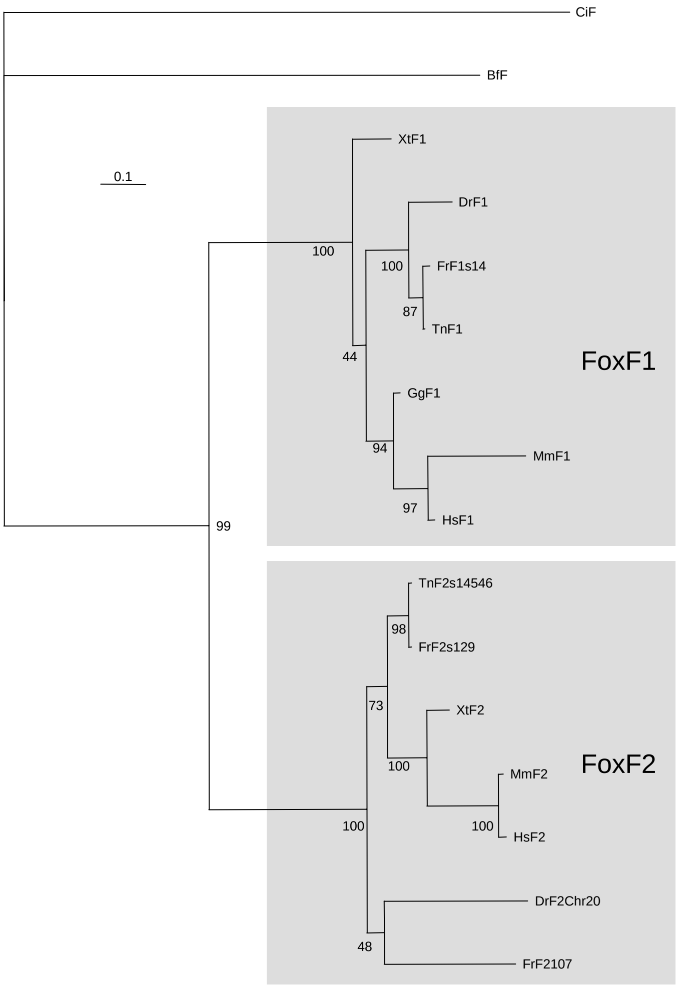

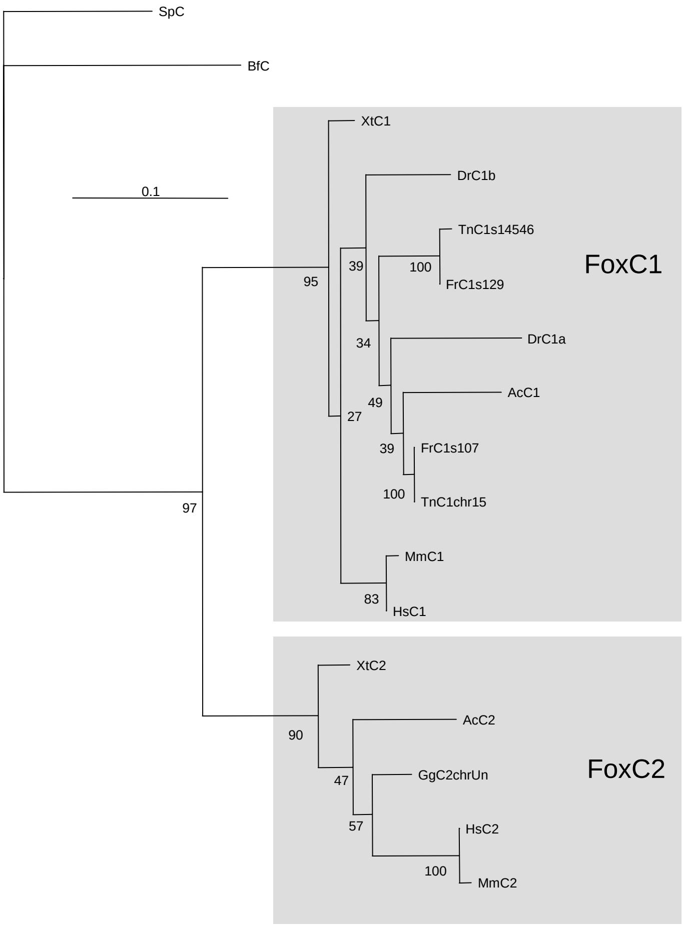

Dusp family maximum likelihood tree

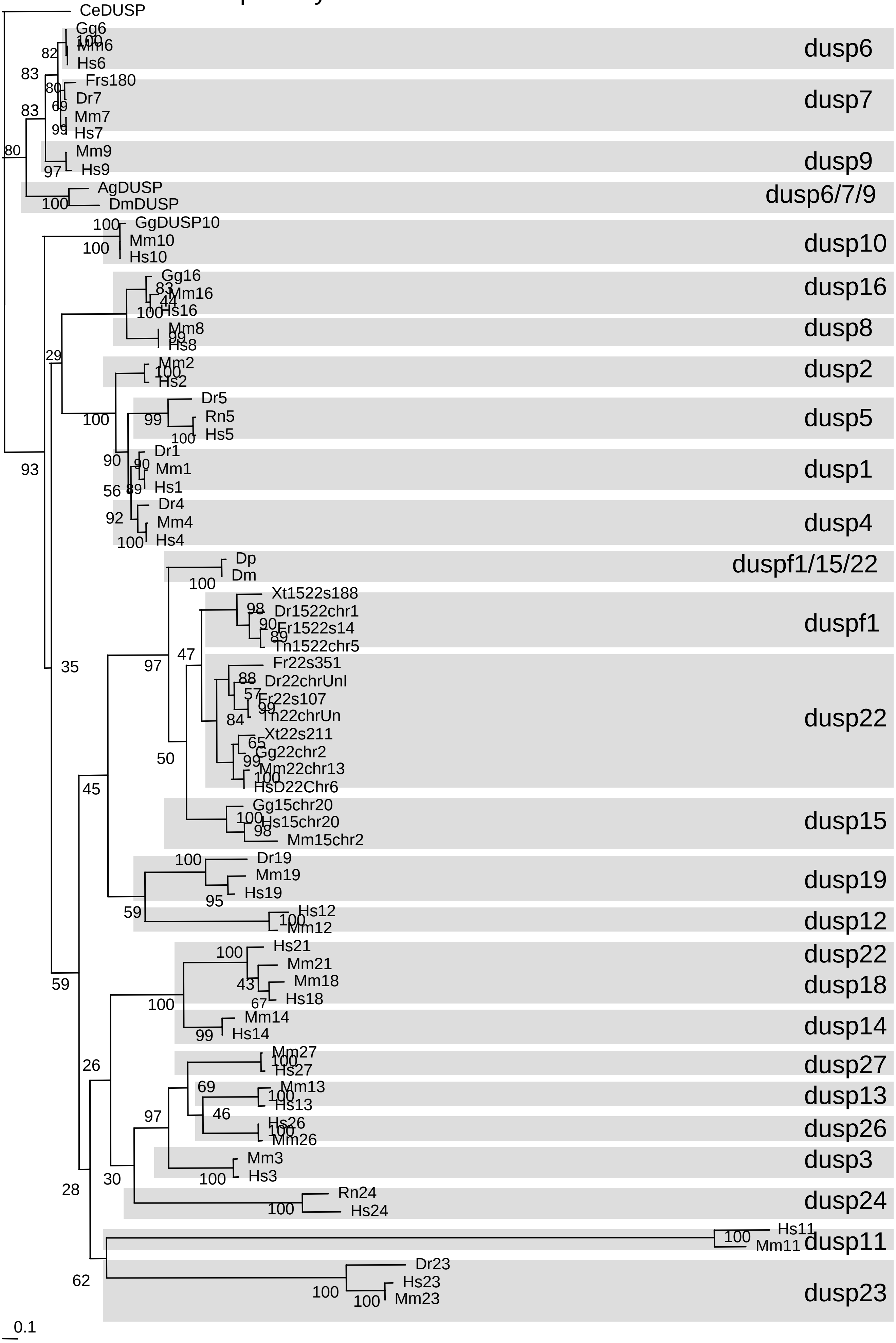

Dusp22, Dusp15 and *Duspf1* subfamily maximum likelihood tree

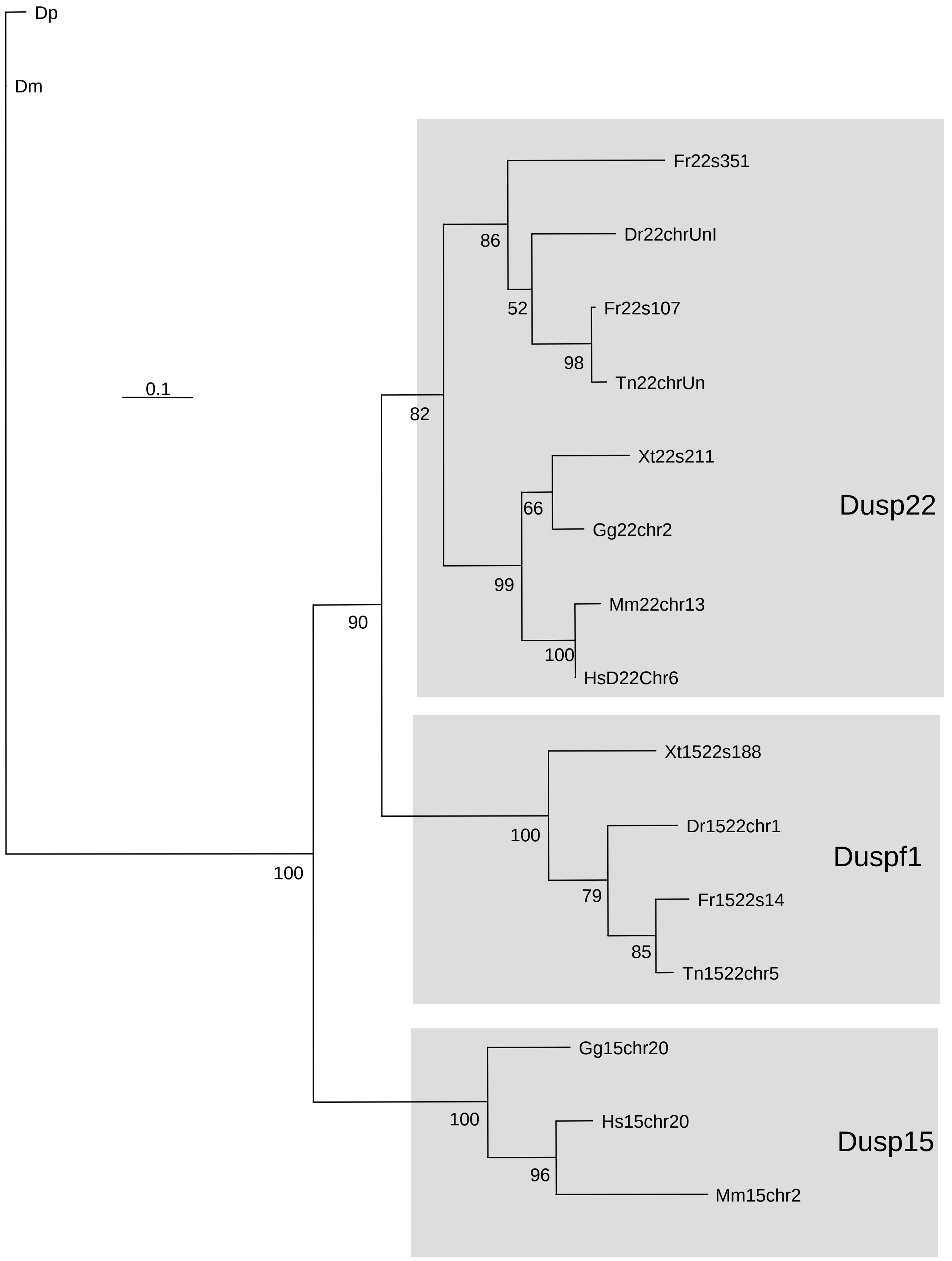

Supplement: Additional File 2 — Phylogenetic trees. Phylogenetic trees of the Fox, MLCK, DUSP, COX, NOC and IRF families used in this analysis. [file 1471-2164-7-271-S2.pdf]
